# Ultrasound-Assisted Extraction of Chickpea Proteins and Their Functional and Technological Properties

**DOI:** 10.17113/ftb.62.04.24.8502

**Published:** 2024-12

**Authors:** Emine Aytunga Arik Kibar, Özlem Aslan

**Affiliations:** TÜBİTAK MAM, Climate and Life Sciences, Food Technology Research Group, Barış Mah. Dr. Zeki Acar Cad. No:1 P.K. 21, 41470Gebze Kocaeli, Türkiye

**Keywords:** chickpea protein, ultrasound-assisted extraction, protein isolate, process optimisation

## Abstract

**Research background:**

Chickpea is a very good source of protein for the development of protein-enriched plant-based ingredients. Chickpea protein isolates are primarily obtained by wet extraction methods such as alkaline or salt extraction. The energy input required for the production of chickpea protein isolates can have an impact on both the environment and processing, thus affecting nutritional quality and human health. Therefore, further research is needed to develop mild processing techniques for the isolation of chickpea proteins.

**Experimental approach:**

In this study, with the aim of developing a more efficient and effective method, chickpea proteins were isolated by ultrasound-assisted extraction and the process parameters were optimised using the Box-Behnken design.

**Results and conclusions:**

Under the optimal extraction conditions (solid/solvent ratio 13.42 g/100 mL, pH=8.8, extraction time *t*=10 min, ultrasound amplitude 70 %), the highest extraction yield was obtained, 66.1 % with ultrasound-assisted extraction and 55.1 % with the conventional alkaline method. When comparing the ultrasound-assisted method with the conventional alkaline method, it was found that a higher protein isolation yield was obtained with a 6-fold shorter processing time and a 29-fold lower energy consumption. Moreover, it was found that the water/oil absorption properties of the protein isolate obtained by the ultrasound-assisted method increased and its foaming properties improved.

**Novelty and scientific contribution:**

This research presents a feasible ultrasound-assisted extraction technique for the isolation of chickpea protein, which can then be used as a versatile ingredient in the food industry.

## INTRODUCTION

Chickpea is the most widely grown pulse crop after beans and peas, accounting for 15 million tonnes of global production. Türkiye is the second largest chickpea-producing country after India. The chickpea is divided into two types: the kabuli chickpea is characterised by its light colour, large seeds and smooth surface, while the desi chickpea is smaller and darker in colour, and is mainly grown in semiarid climate (*1*, *2*).

Chickpea is considered an excellent source of nutrients due to its high protein, fibre, fat and carbohydrate content. It is also an excellent source of proteins because of its high protein bioavailability, nutritional value and well-balanced amino acid content. Chickpeas are one of the most important raw materials for the production of protein-enriched food consumed by vegetarians and vegans. Furthermore, chickpea proteins have good techno-functional properties such as water solubility and good emulsifying, foaming and gelling properties. Therefore, chickpea is an interesting protein source for the development of protein-enriched plant-based ingredients (*1*, *3*).

Chickpea protein isolates are mainly obtained by wet extraction methods such as alkaline or salt extraction. In brief, the chickpea is ground and the resulting flour is defatted to improve extraction performance. The defatted flour is then solubilised in alkaline solution with pH>8.5 so that the protein fraction dissolves in the liquid part. After separation of the non-solubilised non-protein fractions by centrifugation, the solubilised protein fraction is obtained by precipitation at the isoelectric point (*2*).

Ultrasonic technology uses sound waves at the frequency of 20 to 100 kHz, which are generally used in food processing. It induces cavitation, which increases the porosity of the matrix by triggering the formation of small cracks and tunnels on the object that facilitate the penetration of the solvent into the material. The advantages of ultrasound-assisted extraction (UAE) are more effective mixing, faster energy transfer, smaller equipment size and shorter processing time (*4*). Numerous studies have also shown that using ultrasound as a pretreatment increased protein yield or protein release rate. Several ultrasound- and sample-related process factors affect the efficacy of the ultrasound (*5*). The energy density, ultrasound intensity and treatment time are the ultrasound-related factors, while the type of sample/protein and sample/solvent ratio are sample-related parameters (*4*, *6*).

Apart from all the accomplishments and breakthroughs in the use of ultrasound technology in the study of plant-based proteins, it poses challenges for various protein sources and process parameters (*7*-*9*). Therefore, optimisation based on the different plant sources is needed. In addition, high energy consumption is considered a limitation for the use of UAE on an industrial scale. This study aims to optimise the UAE parameters (ultrasound intensity, treatment time, pH, sample/solvent ratio) of chickpea proteins to maximise the process yield using response surface methodology (RSM). In addition, the energy consumption under the optimised extraction conditions was calculated and compared with the energy required for conventional alkaline extraction (CAE). The technological and functional properties of chickpea protein isolates obtained by UAE and CAE methods are also compared.

## MATERIALS AND METHODS

### Materials

The Koçbaşı (Kabuli) chickpea cultivar was purchased from a market in Kocaeli, Türkiye. Analytical grade reagents and solvents from Sigma-Aldrich, Merck (St. Louis, MO, USA) were used.

### Composition of chickpea

Moisture was analysed according to AOAC method 925.09 (*10*). Samples were weighed and dried in an oven (UN55; Memmert GmbH, Schwabach, Germany) at 110 °C for 6 h. After drying, the samples were weighed and the mass loss was reported as mean value of duplicate analyses. Ash content was determined gravimetrically according to AOAC method 923.03 (*11*). The samples were weighed, burned at 600 °C, cooled in a desiccator and the remaining ash was weighed.

### Ultrasound-assisted chickpea protein extraction

Chickpeas were ground with a laboratory mill (SD.06; Empero, Konya, Türkiye) and sieved through 18-mesh to obtain the flour. The chickpea flour was defatted with 1:5 *(m/V*) ethanol for 30 min and dried at ambient temperature by natural convection for 24 h. The defatted chickpea flour was used for protein extraction. An ultrasonic processor UIP2000hdT model (2000 W; Hielscher Ultrasonics GmbH, Teltow, Germany) was used. Four variables were used: sample/solvent ratio (10–20 g/100 mL), pH (8.5–10.5), amplitude (70–90 %) and extraction time (10–20 min). The limit values of the variables were selected based on the preliminary results. Distilled water was used for the extraction and the pH of the mixture was adjusted to 8.5–10.5 with 1 M NaOH. The extraction was carried out at room temperature ((23±2) °C) using a double-walled, cold water circulating tank (MX07R-20/A12E; PolyScience, Nilles, IL, USA). The pH of the supernatant was adjusted to 4.5 with 1 M HCl for protein precipitation. The precipitated protein was isolated using a centrifuge (Universal 320; Hettich, Tuttlingen, Germany) at 7000×*g* for 20 min and rinsed with distilled water. The chickpea protein was then lyophilised (LYO40-ISF Series; Infitek, Spokane, WA, USA) under the following conditions: freezing at -35 °C, main drying at -10 °C, final drying at 25 °C under vacuum (28 kPa) for 65 h and storage at 4 °C for later analysis. For comparison, conventional alkaline extraction (CAE) was carried out under the same conditions as optimal ultrasound-assisted extraction (UAE) by magnetic stirring at 100 rpm for 60 min at a sample/solvent ratio of 13.4 g/100 mL and pH=8.8. The protein content (N×6.25) of each extract was determined using the Kjeldahl method. Protein yield (*Y*/%) was calculated as follows:


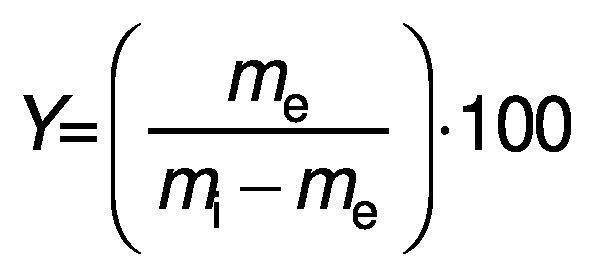
 /1/

where *m*_e_ is the mass of protein in the extract (g) and *m*_i_ is the initial mass of protein (g) in the flour.

### Experimental design

The Box-Behnken design (BBD) was applied using response surface methodology (RSM) to analyse the effects of four independent variables: sample/solvent ratio (A), pH (B), extraction time (C) and percentage of ultrasonic amplitude (D) on protein yield (g extract per 100 g sample) as a response. The experimental runs for BBD and the results are shown in Table 1.

**Table 1:** Box-Behnken design parameters and response

Sample no.	A (*m*(sample)/*V*(solvent))/(g/mL)	B pH	C *t*(extraction)/min	D Ultrasonic amplitude/%	Response *Y*(protein)/%
1	10	8.5	15	80	61.49
2	20	8.5	15	80	50.97
3	10	10.5	15	80	63.49
4	20	10.5	15	80	49.24
5	15	9.5	10	70	61.70
6	15	9.5	20	70	66.66
7	15	9.5	10	90	53.51
8	15	9.5	20	90	64.56
9	10	9.5	15	70	64.59
10	20	9.5	15	70	51.48
11	10	9.5	15	90	63.10
12	20	9.5	15	90	54.81
13	15	8.5	10	80	59.25
14	15	10.5	10	80	56.02
15	15	8.5	20	80	61.20
16	15	10.5	20	80	56.14
17	10	9.5	10	80	61.14
18	20	9.5	10	80	57.88
19	10	9.5	20	80	65.91
20	20	9.5	20	80	56.39
21	15	8.5	15	70	60.92
22	15	10.5	15	70	54.58
23	15	8.5	15	90	56.12
24	15	10.5	15	90	55.99
25	15	9.5	15	80	62.90
26	15	9.5	15	80	58.89
27	15	9.5	15	80	59.62
28	15	9.5	15	80	64.77
29	15	9.5	15	80	62.70

The mathematical model was fitted to the cubic regression equation using Design-Expert software, v. 12 (*12*). The optimal conditions were predicted with RSM and validated by repeated analysis at that point.

### Determination of energy consumption

The energy consumption was calculated according to the following equation:


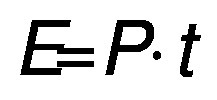
 /2/

where *E* is the energy (J), *P* is the power input (W) and *t* is the time of exposure (s). The energy was divided by the extraction volume (*V*/L) to calculate the specific energy (*E*_s_/(kJ/L)):


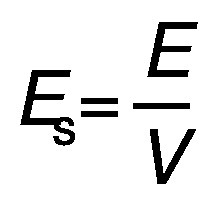
 /3/

The consumed energy (*E*_c_/(kJ/g)) was calculated by dividing the specific energy with the concentration of the protein in the extract (*γ*/(g/L):


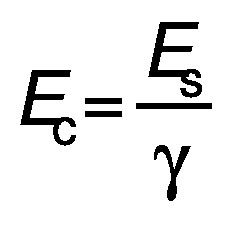
 /4/

### Technological properties of chickpea protein

#### Solubility

Protein dispersions (10 g/L) were prepared at different pH (2–10) using 1 M HCl or 1 M NaOH and mixed for 1 h at 25 °C. Then they were centrifuged (Universal 320; Hettich) at 5000×*g* for 5 min and solubility (in %) was calculated according to the following equation:


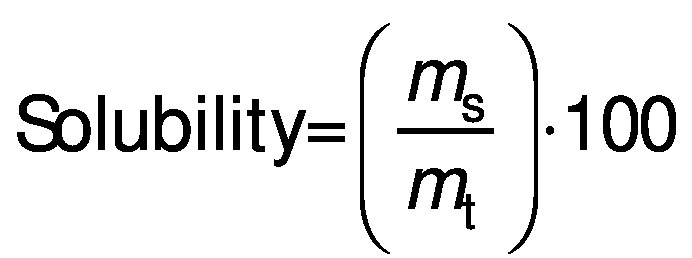
 /5/

where *m*_s_ is the mass of protein in the supernatant (g) and *m*_t_ is the mass of total protein.

#### Oil and water holding capacity

A volume of 3 mL of vegetable oil or deionised water was added to 100 mg of protein concentrate in a 5-mL tube and held at 30 °C for 10 min. The mixture was then centrifuged (Universal 320; Hettich) at 5000×*g* for 10 min, the supernatants were collected and the oil and water absorption capacity of the samples was expressed as the mass of oil or water absorbed per g of sample.

#### Foaming capacity and foam stability

A volume of 15 mL protein dispersion at a concentration of 10 g/L and pH=7.0 was homogenised (Ultra Turrax T25; IKA Werke GmbH & Co KG, Staufen, Germany) at 11 830×*g* for 1 min. Then, the entire content was poured into a 50-mL measuring cylinder and the foaming capacity (FC/%) and foam stability (FS/%) were calculated according to the following equations:


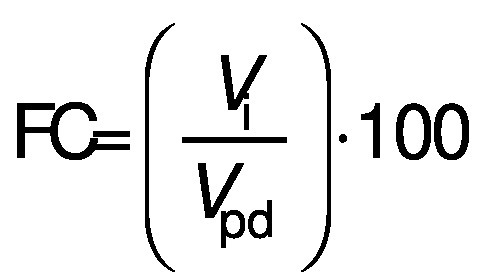
 /6/


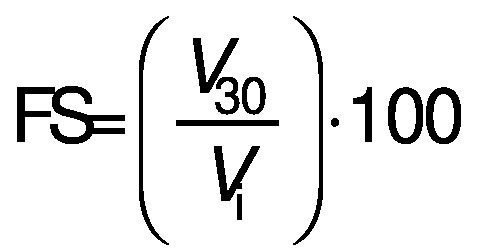
 /7/

where *V*_i_ is the initial foam volume (mL), *V*_pd_ is the volume of protein dispersion (15 mL) and *V*_30_ is foam volume after 30 min.

#### Emulsifying capacity and emulsion stability

A volume of protein dispersion of 15 mL at a concentration of 10 g/L and 5 mL of vegetable oil were homogenised at 13 720×*g* for 3 min (Ultra Turrax T25; IKA Werke GmbH & Co KG). Then 50 µL of the mixture were collected from the bottom and quickly poured to a tube containing 5 mL of 0.1 % sodium dodecyl sulfate (SDS) solution. The absorbance read at 500 nm (spectrophotometer model Lambda 35 UV/Vis; Perkin Elmer Instruments, Waltham, MA, USA) was used for the calculation of the emulsifying activity index (EAI/(m^2^/g)) and the absorbance read after 15 min was used for the calculation of the emulsion stability index (ESI/%) according to the following equations:


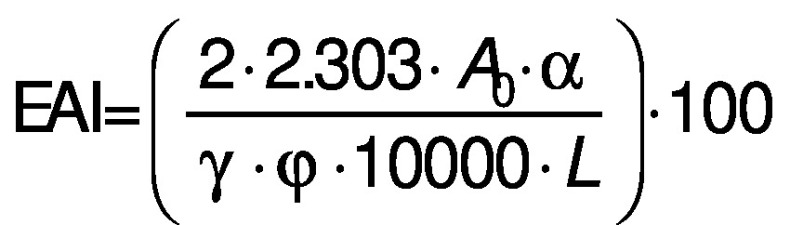
 /8/


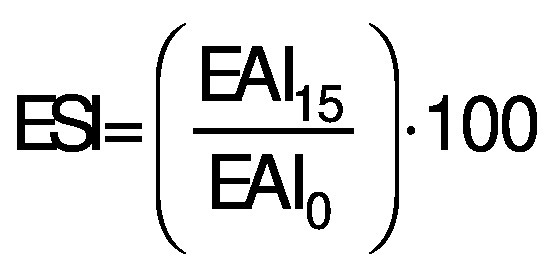
 /9/

where *A*_0_ is the absorbance at 0 min, *α* is a dilution factor, *γ* is the protein concentration (g/mL) and *φ* is the volume fraction of oil.

### Determination of amino acid content

The amino acid content was measured according to Wang *et al.* (*7*) with some modifications. Briefly, 0.5 g homogenised sample was hydrolysed by adding 20 mL of 6 M HCl solution and placed in an oven at 110 °C for 24 h. After derivatisation of the sample, amino acid composition was determined at 254 nm with an ultra-performance liquid chromatography system (LC-20; Shimadzu, Kyoto, Japan) equipped with a UV detector and ACE 5 C18 column (250 mm×4.6 mm, 5 μm) maintained at 40 °C. Gradient elution was used in the system where solvent A (pH=6.9) was prepared with 5 mM NaH_2_PO_4_·2H_2_O and solvent B was analytical grade acetonitrile.

### ATR-FTIR of protein isolates

The spectra were recorded by attenuated total reflectance-Fourier transform infrared spectroscopy (ATR-FTIR; Perkin Elmer). Resolution was 4.0 cm^-1^ and wavenumber accuracy ±0.5 cm^-1^. Spectra (16 scans) were obtained at 4000-600 cm^-1^.

### SDS-PAGE of protein isolates

The method of Laemmli (*13*) for sodium dodecyl sulfate polyacrylamide gel electrophoresis (SDS-PAGE) was followed with a few modifications. At 80 V, the stacking gel (5 %) and the separating gel (15 %) were run (PROTEAN II; BIO-RAD, Hercules, CA, USA). Standards comprised pre-stained protein markers with a molecular mass between 15 and 180 kDa.

### Thermal properties

Differential scanning calorimeter (DSC) model Q20 equipped with Universal Analysis 2000 software v. 4.1D (TA Instruments, New Castle, DE, USA) was used to measure thermal properties. The samples (2–3 mg) were weighed into an aluminium pan and 6–9 μL deionised water were then added to obtain the extract/water ratio of 1:3 (*m/V*). DSC pans were allowed to stabilise at room temperature overnight. The DSC scan was conducted from ambient temperature to 150 °C under the nitrogen gas (30 mL/min). Onset (*T*_o_), peak (*T*_p_) and completion temperatures (*T*_c_) of denaturation endotherm and enthalpy (Δ*H*) were computed from thermograms. The results of duplicate data points were presented.

### Statistical and data analysis

The Box-Behnken experimental design was used for optimisation. Response surfaces and contour plots were plotted using Design-Expert software, v. 12 (*12*) with one independent variable held constant. For each dependent variable, a mathematical model was created using the multiple regression analysis method and the important terms in the model were determined using ANOVA. The statistical evaluation of the results was also carried out using Design-Expert software, v. 12 (*12*). The effect of the factors on the results was determined by the analysis of variance and multiple comparisons. Technological properties were evaluated using one-way ANOVA with *post-hoc* Tukey’s test, which was performed with SPSS v. 11.5 (*14*).

## RESULTS AND DISCUSSION

### Model fitting and method optimisation for UAE

The modified cubic model for the extraction yield of chickpea proteins was fitted according to the Box-Behnken experimental design using Design-Expert software, v. 12 (*12*). The validity of the model was assessed by ANOVA (Table 2). In general, the test for lack of fit resulting in a high F-value and a low p-value (p<0.05) indicates a significant effect of each term on the response parameter. In addition, a high value of the determination coefficient (R^2^) indicates that the model fits the experimental data very well, and a high value of the adjusted determination coefficient (R_adj_^2^) indicates a good fit to the number of independent variables in the model. The predicted coefficient of determination (R_pre_^2^) was used to determine how well a regression model makes predictions. The difference between R_adj_^2^ and R_pre_^2^ was less than 0.2, indicating that the mathematical model is suitable for explaining the experimental data and the high agreement between the predicted and experimental data (*15*). The coefficient of variance (CV/%) lower than 10 % also indicates good precision, reliability and reproducibility of the experimental data (*16*).

**Table 2:** ANOVA results of cubic model fitting

Source	SS	df	Mean value	F-value	p-value	Significance
Model	589.74	18	32.76	10.87	0.000251	**
A	266.46	1	266.46	88.44	0.000003	**
B	27.24	1	27.24	9.04	0.013190	*
C	64.12	1	64.12	21.28	0.000961	**
D	26.44	1	26.44	8.78	0.014228	*
AB	3.50	1	3.50	1.16	0.306740	ns
AC	9.79	1	9.79	3.25	0.101554	ns
AD	5.81	1	5.81	1.93	0.194939	ns
BD	9.66	1	9.66	3.21	0.103619	ns
CD	9.26	1	9.26	3.07	0.110100	ns
A^2^	28.46	1	28.46	9.45	0.011775	*
B^2^	104.48	1	104.48	34.68	0.000153	**
D^2^	8.40	1	8.40	2.79	0.125915	ns
A^2^B	9.76	1	9.76	3.24	0.102022	ns
A^2^C	20.28	1	20.28	6.73	0.026760	*
A^2^D	18.39	1	18.39	6.10	0.033090	*
AC^2^	17.75	1	17.75	5.89	0.035643	*
B^2^C	24.27	1	24.27	8.06	0.017603	*
B^2^D	5.95	1	5.95	1.97	0.190284	ns
Residual	30.13	10	3.01			
Lack of fit	6.07	6	1.01	0.17	0.972276	ns
Std. dev.	1.74				R^2^	0.951
CV/%	2.93				Adjusted R^2^	0.864
Mean value	59.17				Predicted R^2^	0.678

*p˂0.05, **p˂0.01, ns=not significant. SS=sum of squares, A=(*m*(sample)/*V*(solvent))/(g/mL), B=pH, C=*t*(extraction)/min, D=ultrasonic amplitude/%, CV=coefficient of variance

The extraction yield (*Y*/%) was calculated using the cubic polynomial regression equation for the sample/solvent ratio (A), pH (B), extraction time (C) and ultrasonic amplitude percentage (D). The four parameters of UAE had a significant effect on the extraction efficiency in terms of extraction yield (Table 2). The following equation gives the optimised model expressed as coded values:

*Y*=62.2−5.77A−1.85B+4.00C−2.57D−0.93AB−1.56AC+1.21AD+1.55BD+1.52CD−2.06A^2^+3.94B^2^−1.12D^2^+1.91A^2^B −3.18A^2^C+3.03A^2^D+2.58A^2^C−3.48B^2^C+1.72B^2^D /10/

The ANOVA showed that R^2^, R_adj_^2^ and R_pre_^2^ were 0.951, 0.864 and 0.678, respectively. These results show that the model was correctly interpreted for the test data and that the data were in good agreement with the expected values. The yield model was highly significant with a very low p=0.00025. The lack of fit was not significant at p=0.972, indicating that the test data were correctly described by the model. The coefficient of variance was relatively low (2.93 %), indicating that the test results were acceptable (Table 2).

All four linear terms (A, B, C and D), the quadratic terms (A^2^, B^2^ and AB) and the third order terms (A^2^C, A^2^D, AC^2^ and B^2^C) significantly affected the yield (p<0.05) (Table 2).

**Fig. 1:**
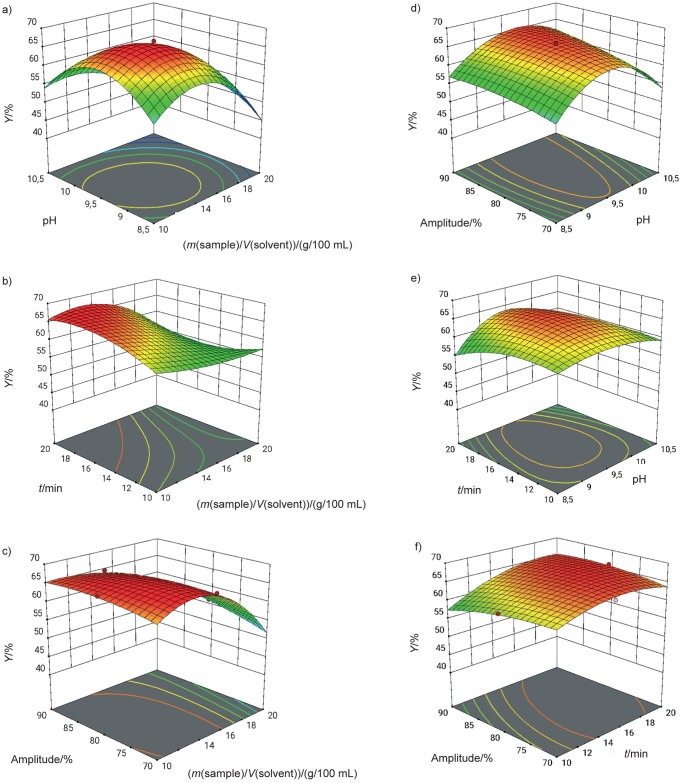
3D response surface plots showing the interactive effects of the independent variables: a) sample/solvent ratio (A) and pH (B) at 70 % amplitude and 20 min extraction time, b) sample/solvent ratio (A) and time (C) at 70 % amplitude and pH=9.5, c) sample/solvent ratio (A) and ultrasonic amplitude (D) at pH=9.5 and 20 min, d) ultrasonic amplitude (D) and pH (B) at a sample/solvent ratio of 10 mg/100 mL and 20 min, e) time (C) and pH (B) at a sample/solvent ratio of 10 mg/100 mL and 70 % amplitude and f) ultrasonic amplitude (D) and time (C) at a sample/solvent ratio of 10 mg/100 mL and pH=9.5

As shown in Fig. 1, three-dimensional response surface plots were generated to evaluate the interaction of the independent variables on the yield. In each plot, one variable was held constant and the other two were changed. The Design-Expert software, v. 12 (*12*) used the desirability function to determine the most effective conditions for ultrasonic extraction. Several solutions were obtained using the desirability function method and the most desirable option with a desirability value of 1 was selected for this study. These were: solid/solvent ratio 13.42 g/100 mL, pH=8.8, extraction time *t*=10 min and ultrasound amplitude 70 %, where the maximum predicted protein extraction yield was 62.8 %. The experimental protein extraction yield was 66.1 %, which corresponded to the value predicted by the response model and thus confirmed its validity.

As shown in Fig. 1a, Fig. 1b and Fig. 1c, the solid/liquid ratio (A) initially had an increasing and then a decreasing effect on the extraction yield. In other words, the extraction yield was higher at the medium solid/liquid ratio than at the minimum and maximum ratios. The distribution of ultrasound-generated energy in the medium and the principles of mass transfer can both be used to explain this. The extraction yield increased with the increase of the solid/liquid ratio from 10 g/100 mL to 14–15 g/100 mL because the difference in the concentration between the extraction liquid and the biomass also increased, which acts as a driving force for mass transfer. On the other hand, the extraction yield decreased with the increase of the solid/liquid ratio from 15 to 20 g/100 mL (Fig. 1a). This can be attributed to the fact that the ultrasound energy applied per unit of extraction volume decreased with the increase in the solid/liquid ratio, which reduced the favourable effect of ultrasound on the extraction efficiency. This is also confirmed by the 3D time-concentration graph in Fig. 1b. Specifically, the extraction time had a variable effect on the yield depending on the solid/liquid ratio. Due to cavitation, mechanical agitation and heat effects, ultrasound facilitates the release of proteins from the biomass into the extraction liquid. As a result, the yield should improve with prolonged ultrasonic application. Low solid/solvent values show the expected trend in this direction. The extraction yield increased from 62 to 64 % when the time was extended from 10 to 20 min. However, with increasing time and a high solid/solvent ratio, the yield decreased from 56 to 52 %. (Fig. 1b). This can be explained by the prolonged ultrasonic treatment, which caused protein degradation and yield reduction.

Another variable that has a significant influence on the yield was the pH. The hydrogen bonds in the biomass are broken by the alkaline solvent, which also improves the efficiency of the extraction. As a result, the extraction yields also increase when the pH increases. Protein degradation decreased the yield when the pH value increased to 9.5–10.5.

### Comparison of energy efficiency

The specific energy values of the two extraction methods were calculated to be 1282 and 27 000 kJ/L for UAE and CAE using Eq. 2 and Eq. 3, respectively. These values represent the energy consumed per unit of extraction volume. Subsequently, the values for the energy consumption for obtaining 1 g protein concentrate were calculated according to Eq. 4, where the final concentrations of the extracts in UAE and CAE were 19.36 and 13.91 g/L, respectively. The energy consumption for 1 g protein concentrate was 66.20 kJ/g (0.02 kWh/g) for UAE and 1941 kJ/g (0.54 kWh/g) for CAE. This result shows that the CAE method consumed roughly 29 times more energy to obtain 1 g of protein than the UAE. This clearly indicates that the UAE is more environmentally friendly and economical than the CAE, as it consumes less energy and has a shorter processing time.

### Effect of extraction method on structural properties of chickpea protein

#### Chemical composition of protein isolates

The maximum protein extraction yield of UAE under the optimal extraction conditions (solid/solvent ratio 13.42 g/100 mL, pH=8.8, *t*(extraction)=10 min, ultrasound amplitude 70 %) was 66.1 %, while the extraction yield of CAE under the same conditions, except for the *t*(extraction)=60 min, was 55.1 %. Under these conditions, the protein content was (86.1±0.2) g/100 g in the isolate obtained with UAE and (99.8±0.1) g/100 g in the isolate obtained with CAE. In this case, extraction with UAE leads to a higher yield but a lower purity of the obtained protein. During the application of UAE, cavitation, agitation and thermal effects can cause the formation of cell wall polysaccharides and protein-polysaccharide conjugates, which become soluble and pass into the extract. Therefore, the content of the protein isolate obtained with UAE was analysed and it was found to contain 1.2 g/100 g starch, 1.3 g/100 g soluble dietary fibre and 3.3 g/100 g insoluble dietary fibre in addition to protein. It was believed that these components affect both the technological and functional properties of the obtained protein isolate.

#### Molecular mass distribution of protein isolates

**Fig. 2:**
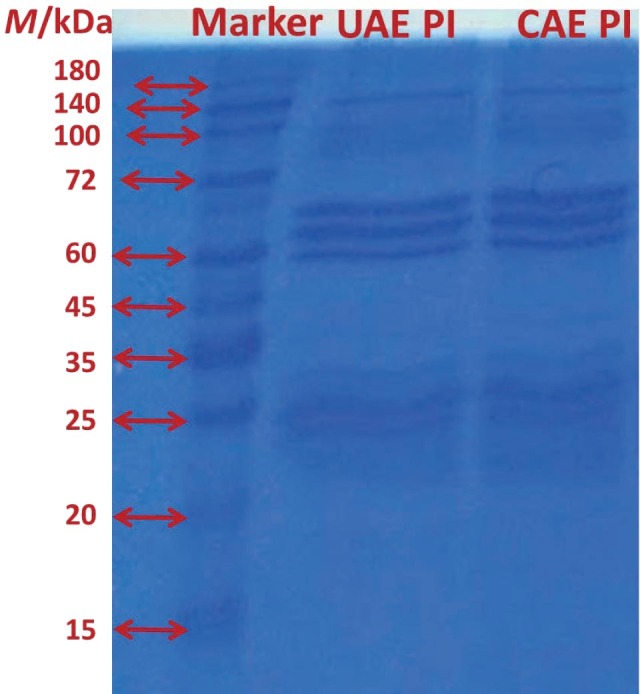
Sodium dodecyl sulfate-polyacrylamide gel electrophoresis (SDS-PAGE) profiles of protein isolates (PI) obtained by ultrasound-assisted extraction under optimal conditions (UAE-PI) and conventional alkaline extraction (CAE-PI)

The content of primary subunits of the chickpea proteins remained unchanged with both extraction methods, as shown by the electropherograms of protein isolate samples (Fig. 2). This indicates that ultrasonic treatment did not cause the break of disulfide links or fragmentation of protein subunits in chickpea proteins. The protein isolates showed two bands at 140 and 100 kDa, which represent albumin protein fractions. The bands between 65 and 72 kDa correspond to convicilin, bands between 58 and 60 kDa correspond to legumin α and β fractions, and very intense bands between 35 and 25 kDa correspond to the legumin α subunits. Similar results were found for chickpea proteins (*17*) and pea proteins (*7*).

#### Amino acid profile of protein isolates

Glutamic acid was the most abundant amino acid in the chickpea protein isolates, followed by aspartic acid and lysine (Table 3). In addition, the mass fractions of the essential amino acids (Trp, His, Phe, Ile, Leu, Lys, Met, Thr and Val) in the chickpea protein isolates were 41.3 and 39.5 g/100 g in the UAE and CAE extracts, respectively. The mass fraction of methionine was 0.65–0.68 g/100 g, confirming the low content of sulphur-containing amino acids (*7*, *17*). Statistically significant differences were found for ten amino acids in the protein isolates obtained by UAE and CAE, with the relative amounts of Ser, Arg, Pro, Leu and Lys being higher in the UAE isolates than in the CAE isolates (Table 3). It was concluded that UAE enables high-quality protein isolates with a higher mass fraction of total amino acids, while achieving a similar amino acid composition.

**Table 3:** Amino acid composition of chickpea protein isolates (CPI) extracted by ultrasound-assisted extraction (UAE-CPI) and conventional alkaline extraction (CAE-CPI) methods

	Extraction method	
(*m*(amino acid)/*m*(protein))/(g/100 g)	UAE-CPI CAE-CPI	
Aspartic acid (Asp)	(11.86±0.09)**^§^**	(12.29±0.05)
Glutamic acid (Glu)	(17.32±0.09)**^§^**	(18.33±0.02)
Serine (Ser)	(4.52±0.03)**^§^**	(4.28±0.03)
Glycine (Gly)	(3.63±0.01)	(3.09±0.03)
Histidine (His)	(2.98±0.04)**^§^**	(3.01±0.02)
Arginine (Arg)	(7.43±0.09)**^§^**	(7.15±0.05)
Threonine (Thr)	(2.55±0.03)	(1.98±0.01)
Alanine (Ala)	(3.93±0.05)	(3.42±0.02)
Proline (Pro)	(4.35±0.04)**^§^**	(4.18±0.01)
Tyrosine (Tyr)	(2.75±0.01)	(2.33±0.02)
Valine (Val)	(4.11±0.04)	(3.62±0.01)
Methionine (Met)	(0.75±0.02)	(0.68±0.01)
Isoleucine (Ile)	(4.49±0.05)	(4.11±0.01)
Leucine (Leu)	(7.61±0.07)**^§^**	(7.22±0.01)
Phenylalanine (Phe)	(6.16±0.07)**^§^**	(6.39±0.01)
Lysine (Lys)	(11.95±0.09)**^§^**	(11.66±0.06)
Tryptophan (Trp)	(0.69±0.01)**^§^**	(0.79±0.01)
Essentials*	41.30	39.46
Non-essentials	55.79	55.06
Total	97.09	94.52

Results are expressed as mean value±standard deviation. *His, Thr, Val, Met, Ile, Leu, Phe, Lys and Trp. ^§^Mean value of amino acids differs significantly (p<0.01) between the chickpea protein fractions obtained by UAE and CAE

#### Thermal stability and denaturation properties of protein isolates

**Fig. 3:**
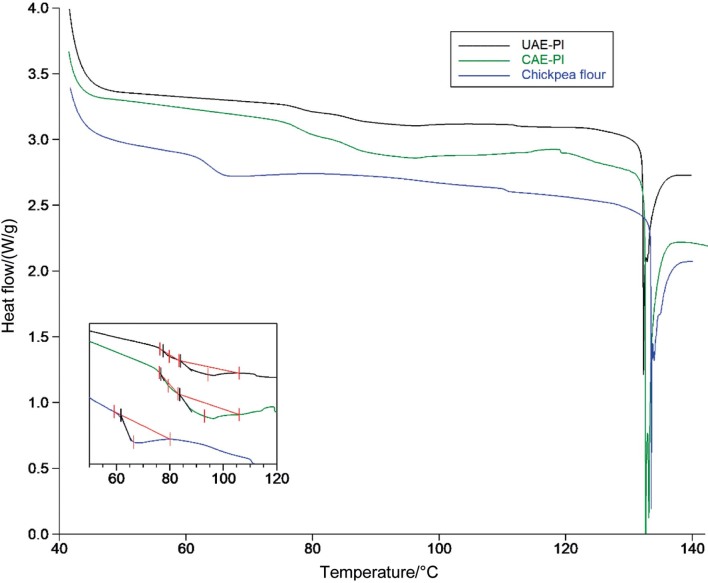
DSC thermograms of chickpea protein isolates (CPI) obtained by ultrasound-assisted extraction under optimal conditions (UAE-PI), conventional alkaline extraction (CAE-PI) and defatted chickpea flour (inset view shows the calculation limits for areas of endothermic peaks)

Differential scanning calorimetry was used to determine the thermal stability of chickpea protein isolates in the presence of excess water (3 g water per g dry mass of extract) to stimulate food processing conditions. The thermal stability of proteins is reflected in their resistance to aggregation in response to heating. Since they can be associated with the heat-induced aggregation and gelation, the thermal properties of globular proteins are very important (*18*). The DSC thermograms (Fig. 3) of both protein isolates show a two-step endothermic transition, which is clearly due to the heat-induced thermal denaturation of the chickpea proteins (*18*–*20*). The overlapping endothermic peaks observed in the DSC thermograms can be attributed to two types of globulins: vicilin and legumin (*20*). The quaternary structure of legumin proteins consists of three dimers linked by non-covalent bonds. Disulfide bridges unite the basic and acidic subunits in each dimer. On the other hand, vicilin has a tertiary structure that is maintained by electrostatic, hydrogen and hydrophobic interactions. Legumin is a more heat-stable protein than vicilin due to its stronger covalently bound structure. Accordingly, the denaturation temperatures of vicilin and legumin are 80–85 and 90–100 °C, respectively (*20*). The DSC thermograms revealed two denaturation endothermic peaks for chickpea protein isolates (CPI) obtained with UAE (UAE-CPI) at temperatures of (80.0±0.3) and (94.2±0.2) °C. Similarly, two denaturation endotherm peaks for chickpea protein isolates obtained with CAE (CAE-CPI) were detected at (79.4±0.3) and (89.9±0.4) °C. Given these data, the endothermic peaks observed at lower temperatures are attributed to the denaturation transition of vicilin fraction, while the endotherm peaks at higher temperatures are attributed to the legumin fraction. There is no statistically significant difference between the denaturation temperatures of protein isolates obtained by different extraction methods. Denaturation temperature is an indicator of thermal stability and is usually associated with higher content of disulphide bonds and higher acid/base amino acid ratio, variations in protein structure and protein-salt interactions (*18*). Therefore, similar thermal stability values of protein fractions obtained with different extraction methods (Fig. 3) indicate similar physicochemical properties of the extracts, such as the vicilin/legumin ratio and the amino acid composition.

The enthalpy values for vicilin unfolding were (0.20±0.1) and (0.36±0.2) J per g extract of protein isolates obtained with UAE and CAE, respectively. Also, the denaturation enthalpy values of legumin fractions obtained with UAE and CAE were (3.45±0.9) and (5.18±1.1) J per g extract, respectively. These results also show that the legumin fractions in the protein isolates were higher than those of vicilin. The obtained results are in accordance with the observations obtained with SDS-PAGE. As can be seen in Fig. 2, denser bands of the legumin fractions (58–60 and 40–35 kDa) than the bands of the vicilin fraction (65–72 kDa) were observed in the electrophoregrams. Moreover, higher enthalpy values of the denaturation endotherms of vicilin and legumin in CAE-CPI than in UAE-CPI can be explained by the higher protein purity of the extract obtained with the CAE method. UAE-CPI contains 86 % protein and a significant amount of non-protein components (starch and dietary fibre), which could explain the relatively lower protein content of the extract obtained with UAE than of those obtained with CAE. Defatted chickpea flour was used to compare the thermal behaviour of the matrix containing chickpea proteins and non-protein compounds (starch and dietary fibre) (Fig. 3). The typical starch gelatinisation endotherm was observed between 61 and 77 °C with a peak temperature of 66 °C and enthalpy of 6.88J/g. It can be said that the component that has the greatest influence on the thermal behaviour of chickpea flour is the starch and the denaturation peak was not observed due to the low protein content.

#### FTIR characterisation of protein isolates

**Fig. 4:**
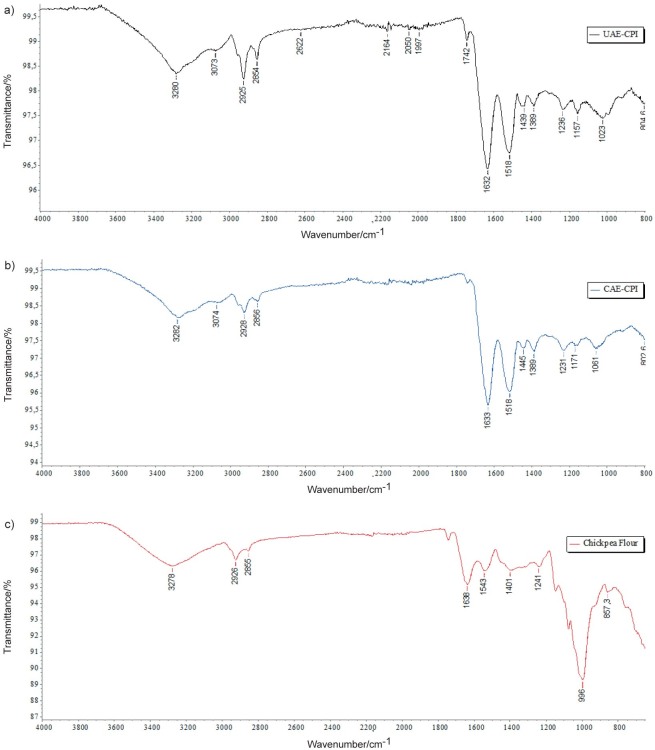
Attenuated total reflectance-Fourier transform infrared (ATR-FTIR) spectra of chickpea protein isolates (CPI) obtained by: a) ultrasound-assisted extraction under optimal conditions (UAE-CPI), b) conventional alkaline extraction (CAE-CPI) and c) defatted chickpea flour

FTIR spectroscopy was used to analyse the chickpea protein isolates obtained in this study to determine any changes in composition that could result from the extraction process and compare to the spectrograph of whole chickpea flour. The FTIR spectra of the protein isolates show typical protein bands (Fig. 4): the primary amide band at around 1632 cm^-1^ is attributed to C=O stretching vibration, the secondary amide band at 1518 cm^-1^ corresponds to N-H in-plane bending and C-N stretching, and the bands between 1439 and 1157 cm^-1^ regions are attributed to tertiary amide, which corresponds to C-N stretching (*19*, *21*–*23*). Moreover, the broad band at about 3400 cm^-1^ was associated with the stretching of hydrogen-bonded hydroxyl groups. The axial deformation of the CH_2_ group is responsible for the intense band at approx. 2930 cm^-1^. In addition, the typical bands of stretching vibrations of C-O bonds in C-O-H and C-O-C groups in the anhydroglucose ring within the polysaccharide are found at 1157 and 1023 cm^−1^ in UAE-CPI and 1171 and 1061 cm^-1^ in CAE-CPI, respectively (*24*). The chickpea flour spectrum reflects the main peaks observed for the protein isolates, as well as the very intense bands between 1150 and 800 cm^-1^ that are attributed to starch and sugars, as previously reported in the literature (*19*).

### Effect of extraction method on technological properties of chickpea protein

#### Solubility of protein isolates

One of the most important functional properties of proteins is their solubility. The lowest solubility values for UAE-CPI and CAE-CPI were observed between pH=4 and pH=6 (Table 4), which is due to the decrease in the net charges of the protein around the isoelectric point (*23*). The molecular size and structure of the proteins are important factors that affect the solubility behaviour. The solubility of CAE-CPI increased to 89.6 % at pH=2 and thus improved compared to UAE-CPI. However, the decreasing effect of ultrasound on particle size and α-helix is expected to increase water-protein interactions and thus increase solubility (*7*). In the present study, the lower protein purity and thus the non-protein substances could cause the lower solubility of UAE-CPI than of CAE-CPI. The protein had more charges when the pH was above the isoelectric point (pH>4.5). This led to an increase in electrostatic repulsion and hydration, which facilitated protein dissolution and increased solubility (Table 4).

**Table 4:** Technological properties of chickpea protein isolates (CPI) extracted by ultrasound-assisted extraction (UAE-CPI) and conventional alkaline extraction (CAE-CPI) methods

	**Extraction method**	
**Property**	UAE-CPI	CAE-CPI
**OHC/(g/g)**	(8.7±0.9)^a^	(7.4±0.4)^b^
**WHC/(g/g)**	(3.1±0.4)^a^	(2.50±0.01)^b^
**FC/%**	(18.3±0.5)^a^	(11.7±0.5)^b^
**FS/%**	(85.4±0.4)^a^	(79.6±0.6)^b^
**EAI/(m^2^/g)**	(75.5±1.8)^b^	(159.3±6.5)^a^
**ESI/%**	(93.3±1.2)^b^	(99.1±1.8)^a^
**pH**	Solubility/%	
**2**	(62.9±3.0)^b^	(87.1±3.6)^a^
**4**	(5.0±1.3)^a^	(3.8±0.1)^b^
**6**	(4.7±1.6)^a^	(4.8±0.6)^a^
**8**	(4.0±2.1)^a^	(4.9±1.9)^a^
**10**	(7.9±2.4)^a^	(8.2±0.6)^a^

Results are expressed as mean value±standard deviation. Mean values with different letters in superscript in the same row differ significantly (p<0.01). OHC=oil holding capacity, WHC=water holding capacity, FC=foaming capacity, FS=foam stability, EAI=emulsifying activity, ESI=emulsion stability index

#### Oil and water holding capacity of protein isolates

A high oil holding capacity (OHC) is favourable for proteins used in meat products, meat substitutes, extenders and bakery products (*25*). Water holding capacity (WHC) is a key functional characteristic of proteins in thickening and viscous foods, like bakery products, because these foods can adsorb water without causing protein disintegration (*26*). Table 4 shows the OHC and WHC values of the protein isolates and the values are consistent with those reported for germinated chickpea proteins (*23*).

As can be seen in Table 4, UAE-CPI has a significantly higher WHC than CAE-PI. Ultrasonication can lead to the formation of smaller particles, which may improve the protein-water interactions and thus increase the water absorption by the resulting isolate. WHC of the isolates obtained in this study (2.5–3.7 g/g) is within the recommended range for proteins used in food processing (1.49-4.72 g/g) (*7*). The WHC values are also higher than those of sunflower meal protein (0.99 g/g) (*26*), rice bran protein (2.59 g/g) (*27*), comparable to pea protein (3.0-4.1 g/g) (*7*) and wampee seed protein (3.93 g/g), but lower than soy protein (5.4 g/g) (*28*).

The OHC values of UAE-CPI were also significantly higher than those of CAE-CPI, *i.e.* the application of ultrasound improved the oil absorption capacity of chickpea proteins (Table 4). This could be due to the interaction with hydrophobic groups induced by ultrasound treatment (*26*). The OHC of UAE-CPI (8.66 g/g) in this study was significantly higher than the values of pea protein (5.8–6.2 g/g) (*7*), sunflower meal protein (2.06 g/g) (*26*), wampee seed protein (3.25 g/g) and soy protein (2.19 g/g) (*28*). This result showed that UAE-CPI can be used in many food formulations where high OHC is crucial.

#### Foaming properties of protein isolates

The higher foaming capacity and foaming stability values of the chickpea protein isolate produced with UAE indicate that the ultrasonic treatment improves the foaming properties of chickpea protein (Table 4). The molecular rigidity of the protein determines the foaming stability, while the foaming capacity is defined as the protein flexibility and sorption amount at the air-water boundary. As a result, improved foaming behaviour induced by ultrasound can be associated with significant changes in protein structure, which increases the adsorption capacity of the protein at the interface (*26*).

#### Emulsification properties of protein isolates

The process of emulsification is crucial in the production of many foods. The amount of oil-water interface stabilised by protein per unit of mass is measured by the emulsion activity index (EAI), while the ability of a protein to maintain emulsion stability over time is assessed by the emulsion stability index (ESI). Table 4 shows the EAI and ESI values of chickpea protein isolates. The protein isolate obtained by the conventional method had a twofold higher EAI than the isolate obtained by the ultrasound-assisted method. This could be attributed to the fact that ultrasound can affect the emulsion properties of chickpea proteins due to the simultaneous extraction of non-protein components, which can limit the emulsifying active sites of the protein. The ESI of both protein isolates are not significantly different, so that both chickpea protein isolates can be used to produce very stable emulsions. The values measured in this study are similar to those reported for chickpea proteins (*29*), but considerably higher than those reported for other protein isolates. The reported EAI and ESI values are: 40.4 m^2^/g and 84 % for brewers’ spent grain protein (*9*), 21.4 m^2^/g and 66 % for pea protein (*7*), 50.7 m^2^/g and 50.4 % for sunflower meal protein (*26*), 77.6 m^2^/g and 96.2 % for wampee seed protein and 56.7 m^2^/g and 85.3 % for soy protein, respectively (*28*). This is an indication of the superior technological properties of chickpea protein among plant proteins.

Consequently, it can be said that ultrasonication may have more advantages in improving many technological properties of chickpea proteins compared to other innovative food processing technologies. Namely, Wang *et al.* (*29*) applied pH shifting, cold plasma treatment and their combination. They found that the combined treatment improved many of the technological properties of chickpea protein isolate. However, the cold plasma treatment alone did not improve emulsion and foaming stability and led to a very limited increase of emulsion activity, foaming capacity, water holding capacity and oil holding capacity. In another study using an innovative method (*30*), an attempt was made to improve the functional properties of chickpea protein using high pressure. The emulsion and foaming properties were improved only when high pressure was applied for more than one cycle.

## CONCLUSIONS

Ultrasonication is a promising technology that is currently being extensively researched for many applications in food processing, particularly for the development of plant-based protein components. In this study, chickpea proteins were isolated using an ultrasound-assisted extraction method. The parameters that significantly affected the extraction yield were the solid/solvent ratio and pH. Under optimum conditions, the yield increased from 55.1 to 66.1 %, the extraction time decreased from 60 to 10 min, the energy input decreased from 1941 to 66.20 kJ per kg protein, but the protein purity of the isolate decreased from 99.8 to 86.1 %. This indicates that ultrasound simultaneously favours the extraction of some non-protein components (starch and dietary fibre). Nevertheless, the application of ultrasound had no significant effect on amino acid composition, molecular mass distribution, protein primary structure or thermal stability. Furthermore, the application of ultrasound improves water and oil retention capacity as well as foaming properties. As a result, ultrasound-assisted extraction of chickpea protein is more efficient, environmentally friendly and cost effective than traditional methods, because it takes less time, uses less energy and does not affect the technological properties of the protein. However, there may be some disadvantages, such as the modification of the ingredients in a liquid medium, which requires subsequent drying to preserve the altered ingredients or to facilitate the preparation of certain food products. Therefore, an economic analysis of ultrasound-assisted ingredient production could be a basis for future research study. Moreover, laboratory-scale ultrasound is predominantly carried out in batch mode, so further research on pilot-scale and/or commercial applications is needed to generalise the practical use of ultrasonication in the food industry, especially in the plant-based proteins.
